# TBX21 inhibits colorectal cancer metastasis through ARHGAP29/GSK3β inhibitory signaling- and MYCT1/ZO-1 signaling-dependent manner

**DOI:** 10.7150/ijbs.97920

**Published:** 2025-01-01

**Authors:** Xiaowen Yang, Xinzhuang Shen, Zongjun Liu, Yifei Li, Hangchu Liu, Yiting Zhan, Yipeng Liu, Chengjin Liang, Luping Zhao, Xiaoyuan Zhang, Yongming Huang, Wenzhi Shen

**Affiliations:** 1 Cheeloo College of Medicine, Shandong University, Jinan, 250012, China.; 2 Shandong Provincial Precision Medicine Laboratory for Chronic Non-communicable Diseases, Institute of Precision Medicine, Jining Medical University, Jining, 272067, China.; 3 Department of Medical Imaging, Affiliated Hospital of Jining Medical University, Jining Medical University, Jining, 272000, China.; 4 Department of General Surgery, Affiliated Hospital of Jining Medical University, Jining Medical University, Jining, 272000, China.

**Keywords:** Colorectal cancer (CRC), TBX21, metastasis, ARHGAP29/GSK3β, MYCT1/ZO-1

## Abstract

T-box transcription factor 21 (TBX21) plays a vital role in regulating immune responses, systemic diseases, and tumor progression. However, the role of TBX21 in colorectal cancer (CRC) metastasis remains unclear. In this study, we observed that TBX21 expression was marked decreased in CRC tissues compared to normal tissues and was negatively correlated with TNM stages. Interestingly, CRC and normal cell lines lacked TBX21 expression. Ectopic expression of TBX21 inhibited CRC cell migration *in vitro* and metastasis *in vivo*. Furthermore, a human phospho-kinase array analysis indicated that TBX21 expression reduced phosphorylation of glycogen synthase kinase 3 beta (GSK3β). RNA sequencing identified Rho GTPase activating protein 29 (ARHGAP29) and MYC target 1 (MYCT1) as potential TBX21-related target genes. TBX21 directly binds to the ARHGAP29 promoter to upregulate ARHGAP29, which, in turn, inhibits GSK3β phosphorylation. Concurrently, TBX21 promotes MYCT1 expression, leading to interaction with ZO-1 to regulate the cytoskeleton. Together, the ARHGAP29/GSK3β and MYCT1/ZO-1 pathways suppress CRC cell metastasis. Conversely, knockdown of TBX21, ARHGAP29, or MYCT1, or LiCl application, which enhances GSK3β phosphorylation, counteracted TBX21-mediated inhibition of CRC cell migration *in vitro* and *in vivo*. These findings suggest TBX21 suppresses CRC metastasis, identifying it as a potential target for metastatic cancer treatment.

## Introduction

As the third most common malignant tumor and the second leading cause of cancer-related mortality 1, colorectal cancer (CRC) represents a substantial threat to human health. Over 50% of patients with CRC present with advanced-stage disease at the time of diagnosis 2, and the 5-year overall survival rate for individuals with stage IV CRC is limited to 10% 3. CRC frequently metastasizes to distant organs, particularly the liver and lungs 4, 5. The spread of cancer cells from the primary tumor to distant sites occurs through a multistep process known as the invasion-metastasis cascade 6. The epithelial-to-mesenchymal transition (EMT) program constitutes a critical phase of this cascade, equipping tumor cells with properties essential for invasion and metastatic progression, including enhanced motility, invasiveness, and the ability to degrade components of the extracellular matrix 7, 8. Despite advancements in CRC diagnosis and therapeutic interventions, 5-year survival rates remain low due to tumor metastasis and recurrence. Therefore, identifying targets related to CRC metastasis and elucidating their underlying mechanisms is of paramount importance.

TBX21 is a member of the T-box family of transcription factors that governs the differentiation and function of diverse immune cell types 9. Extensive studies on TBX21 have extended its expression profile to include immune cells such as B cells, dendritic cells, natural killer cells, innate lymphoid cells, as well as epithelial cells within the reproductive and respiratory systems 10. Emerging evidence indicates that TBX21 is involved in the pathogenesis of various diseases. For example, T-bet-deficient, non-diabetic mice are protected from diabetes 11, and TBX21 is overexpressed in the peripheral blood leukocytes of patients with late-onset Alzheimer's disease 12. Recent findings also highlight a critical role for TBX21 in tumor progression. Specifically, TBX21 deletion in dendritic cells promotes colitis-associated colorectal carcinogenesis 13, and TBX21 has been implicated in lung carcinogenesis via the TBX21/IL-4 signaling pathway 14. In CRC, TBX21 inhibits cell proliferation in an ARHGAP29/RSK/GSK3β-dependent manner 15. However, the specific functions and molecular mechanisms of TBX21 in CRC metastasis remain unclear.

ARHGAP29 (Rho GTPase-activating protein 29) is a PARG1- or PTPL1-related Rho GTPase-activating protein 16. ARHGAP29 contains a GTPase-activating protein (GAP) domain, exhibits a high affinity and inhibitory activity for RhoA, and shows a weak affinity for Rac2 and Cdc42. Recent research has elucidated the role of ARHGAP29 in the progression of various diseases. For instance, in glomerulonephropathy, the YAP/TAZ-ARHGAP29-RhoA signaling axis regulates podocyte protrusion and integrin adhesion 17. The role of ARHGAP29 varies across different tumor types; for example, upregulated ARHGAP29 expression promotes proliferation and invasion of prostate cancer cells 18, and invasive, mesenchymally-transformed breast cancer cells exhibit elevated ARHGAP29 expression 19. In a previous study, ARHGAP29, identified as a downstream target gene of TBX21, was shown to inhibit proliferation and promote apoptosis in CRC cells 15. Nonetheless, the specific role of ARHGAP29 in CRC metastasis remains insufficiently understood.

Glycogen synthase kinase-3β (GSK3β) is a serine-threonine kinase within the glycogen synthase kinase family. GSK3β plays a crucial role in energy metabolism, inflammation 20, endoplasmic reticulum (ER) stress 21, mitochondrial dysfunction 22, and apoptotic pathways 23. Phosphorylation at Ser9 induces a pseudo-substrate conformation in the docking mode of the GSK3β substrate, leading to GSK3β silencing. This modification results in GSK3β inactivation via proteasomal degradation, a process implicated in various pathological conditions, including cancer 24. While the functions and mechanisms of GSK3β are well-documented, its specific role in TBX21-mediated cancer metastasis remains unexamined.

Myc target 1 (MYCT1), located at chromosome 6q25.2, is a candidate tumor suppressor gene initially identified in laryngeal squamous cell carcinoma. MYCT1 is broadly expressed across cell types, though its subcellular localization varies among different cancer types 25. Studies indicate MYCT1 is involved in various disease pathways; for instance, it mitigates renal fibrosis and tubular injury in diabetic nephropathy 26. MYCT1 binds to MAX and may inhibit RUNX1 transcription, thus suppressing proliferation and the cell cycle in diffuse large B-cell lymphoma cells 27. MYCT1 overexpression inhibits proliferation and induces apoptosis in human acute myeloid leukemia HL-60 and KG-1a cells both *in vitro* and *in vivo* 28. MYCT1 expression is downregulated in human gastric cancer cell lines, where it accelerates apoptotic cell death 29. MYCT1 variants are also associated with retinoblastoma 30 and IgA nephropathy 31. However, the specific function of MYCT1 in the progression remains undefined.

In this study, we examined TBX21 expression in CRC cells, investigating the effects of TBX21 overexpression and knockdown on CRC cell migration *in vitro* and metastasis *in vivo*. We conducted a detailed analysis of the potential mechanisms through which TBX21 influences CRC metastasis. Additionally, we validated the TBX21-mediated signaling pathway using LiCl and specific short hairpin RNAs (shRNAs) *in vitro*. Our findings identify novel markers of CRC metastasis and present new targets and strategies for CRC metastasis treatment.

## Materials and Methods

### Online database analysis

The TIMER (Tumor Immune Estimation Resource) online database (http://timer.comp-genomics.org/) and the UALCAN database (https://ualcan.path.uab.edu/) were used to examine TBX21 expression levels in healthy colon and CRC tissues.

### Human CRC tissues

All human CRC tissue samples were obtained from patients undergoing surgery at the Affiliated Hospital of Jining Medical University between 2015 and 2019. Detailed patient information is provided in Table S1. TNM staging was assigned to all tissue samples in accordance with the American Joint Committee on Cancer (AJCC) guidelines 32.

### Immunohistochemical (IHC) staining

The human CRC tissue microarrays were procured from Biotech (cat. #D150Co01, cat. #D068Co02, Xi'an, China), and paraffin-embedded sections were obtained from tumors grown *in vivo*. Immunohistochemical (IHC) staining was conducted following established protocols as previously described 33. The specific antibodies used are listed in Table S2.

### Vector construction

The human TBX21 overexpression vector (pLVML-3×FLAG-TBX21) was obtained from Fenghui Biology Company (Changsha, China). Plasmids containing shRNAs targeting TBX21, ARHGAP29, and MYCT1 were constructed based on previously described protocols. The shRNA oligonucleotides were synthesized, annealed to form double-stranded oligos, and ligated into a linearized pLV-shRNA-puro vector (cat. #B19; Biosettia, USA) to construct circularized pLV-shRNA-puro plasmids 14, 34. All shRNA sequences are summarized in Table S3.

### Cell culture

Both RKO and SW620 cells were utilized for wound-healing assays due to their faster growth rates and suitability for non-aggregated growth patterns, which facilitated scratch formation. RKO cells were cultured in Dulbecco's Modified Eagle Medium (DMEM) (cat. #24X681, Pricella) supplemented with 10% fetal bovine serum (FBS) (cat. #UB68506T050, BIOODIN), 1% sodium pyruvate (Gibco), 1.5 g/L NaHCO3 (Gibco), and 1% non-essential amino acids (NEAA) (Gibco). SW620 cells were cultured in L15 medium (Gibco) supplemented with 10% FBS. All CRC cell lines were obtained from the American Type Culture Collection (ATCC) and initially tested for mycoplasma contamination using a Mycoplasma PCR kit to confirm they were mycoplasma-free.

### Western blotting

Western blotting was performed according to previously established protocols 35. Proteins (10-50 μg) were loaded onto a 4-20% polyacrylamide gradient gel, transferred to a polyvinylidene difluoride (PVDF) membrane, blocked, and then probed sequentially with a primary antibody and a horseradish peroxidase (HRP)-conjugated secondary antibody. The blotting results were visualized using the ECL reagent kit (cat. #BL520A; Millipore, Biosharp, Hefei, China) and a chemiluminescence imager (Tanon, Shanghai, China). All primary antibodies used in this assay are detailed in Table S2.

### Wound healing assay

Cells (5 × 10^5)^ were seeded and grown to 90% confluence. A scratch wound was created using a sterile pipette tip, and images were captured at 0, 12, and 24 hours using an inverted microscope (Olympus, Tokyo, Japan). Three independent experiments were performed.

### Transwell migration assay

CRC cells (2 × 10^5^) were suspended in 1% serum medium and seeded into Boyden chamber inserts with 8 µm pore membranes (Corning Inc., Corning, NY, USA). Medium containing 10% fetal bovine serum (FBS) was added to the lower chamber. After 24 hours, the inserts were fixed, washed, and stained with 0.1% crystal violet. Images were captured for statistical analysis.

### *In vivo* xenograft model

To establish the xenograft tumor model, male nude mice (6-8 weeks old) were randomly assigned to groups (n = 6), and 3 × 10^6^ cells were injected subcutaneously into each mouse. Tumor volume (mm^3^) was calculated as follows: volume (mm^3^) = (width^2^ × length) / 2.

Mice were humanely euthanized using Avertin (Sigma-Aldrich, Shanghai, China) (dissolved in tert-amyl alcohol and 0.9% saline, 0.2-0.4 mL/10 g mouse body weight, intraperitoneal injection). Tumors and lungs excised from the mice were fixed in formalin, embedded in paraffin, and sectioned 36. All animal experiments were performed in accordance with the Jining Medical University Animal Welfare Guidelines and were approved by the Jining Medical University Animal Ethics Committee.

### Hematoxylin and eosin (HE) staining

Mouse lung tissue was fixed in 4% paraformaldehyde for at least 48 hours, then dehydrated, embedded, and sectioned to produce 4 µm-thick paraffin sections. The HE staining procedure involved deparaffinization, rinsing in distilled water, hematoxylin staining of the nuclei, differentiation with hydrochloric acid alcohol, eosin staining of the cytoplasm, and mounting with a medium suitable for long-term storage.

### Human phosphokinase array

Phosphorylation levels of selected kinases and proteins were measured using a Human Phosphokinase Array Kit (cat. # ARY003C, R&D Systems, USA), following the manufacturer's instructions. Cell lysates were prepared by adding 1 mL of lysis buffer to 1 × 10^7^ cells and gently shaking at 2-8°C for 30 minutes. Membranes A and B were incubated in Array Buffer 4 for 1 hour at room temperature for blocking. A mixture of 334 µL of cell lysate and Array Buffer 1 (2 mL total) was incubated with Membranes A and B overnight at 2-8°C. Membrane A was incubated with Cocktail A, and Membrane B with Cocktail B at room temperature for 2 hours on a shaker. Following this, both membranes were incubated with streptavidin-HRP for 30 minutes at room temperature and visualized using an ECL reagent after washing.

### RNA sequence

Three RKO-MCS and RKO-TBX21 cells (1 × 10^6^ each) were separately collected, washed three times with phosphate-buffered saline (PBS), and suspended in 1 mL of TRIzol reagent (Thermo Fisher Scientific, USA). RNA extraction, library construction, RNA sequencing, and data analysis were performed by Biomarker Biologicals (Beijing, China). Data were submitted to the Gene Expression Omnibus (GEO) database (Accession No: GSE226100).

### Immunoprecipitation (IP)

RKO cells overexpressing TBX21 were cultured in a 10 cm dish. Proteins were extracted, and the primary antibody was incubated with the lysate overnight at 4°C. Protein A/G PLUS-agarose was added, followed by incubation at 4°C for 2 hours and centrifugation. The precipitate was washed twice with radioimmunoprecipitation assay (RIPA) buffer, and loading buffer was added. Western blotting was subsequently performed.

### Immunofluorescence (IF)

Cells (2 × 10^5^ cells/well) were cultured in a 24-well plate, washed with PBS, and fixed with 4% paraformaldehyde for 15 minutes at room temperature. Permeabilization was performed with 0.5% Triton X-100 for 15 minutes, followed by blocking with 5% goat serum for 1 hour. Cells were incubated with the primary antibody overnight, followed by the secondary antibody for 1 hour at room temperature. Nuclei were stained with DAPI and visualized under a confocal microscope. Details of specific antibodies and fluorescent secondary antibodies are available in Table S2.

### Cytoskeleton staining

Cells were rinsed with PBS and incubated with 5 μg/mL Actin-Tracker Red-Rhodamine (cat. # sc-2002, Santa Cruz Biotechnology, USA) for 30 minutes at room temperature. Images were acquired using a 40× objective lens on a confocal microscope (Olympus).

### Statistical analysis

Data analysis was performed using GraphPad Prism 5 software (GraphPad Software, San Diego, CA, USA). Values are expressed as the mean ± standard error of the mean (SEM). Statistical significance was determined using Student's t-test for comparisons between two groups or one-way analysis of variance (ANOVA) for comparisons among multiple groups, unless otherwise specified. A p-value of < 0.05 was considered statistically significant.

## Results

### TBX21 expression is downregulated in human CRC tissues

**Figure 1:**
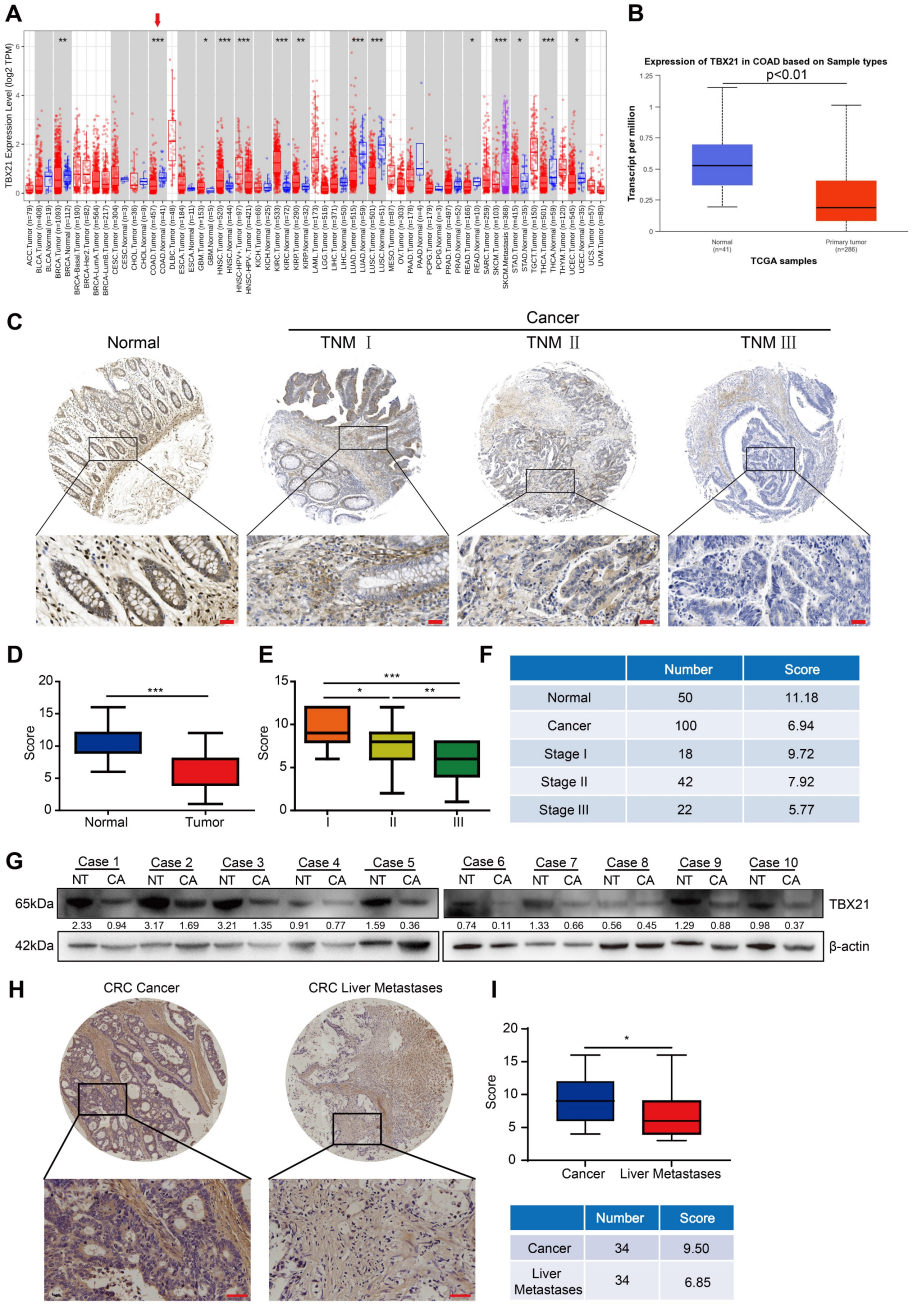
** TBX21 is downregulated in human CRC tissues. A-B.** TIMER and UALCAN online databases were used to analyze the transcriptional expression of TBX21 in colon cancer (cancer vs. normal tissue; A, p<0.05; B, p<0.01). **C.** Representative IHC images of TBX21 in a human CRC tissue microarray. Scale bar: 50 μm. **D.** Quantification of TBX21 expression in the CRC tissue array. ***p<0.001. **E & F.** Quantification of the correlation between TBX21 expression and tumor TNM stage (18 of these tumor patients had missing TNM stage information). *p<0.05, **p<0.01, ***p<0.001. **G.** Western blot analysis of TBX21 expression in fresh colon cancer and adjacent normal tissues, with β-actin as a loading control. NT represents normal tissues; CA represents cancer tissues. The experiment was repeated three times, with data shown from one representative experiment. **H.** Representative IHC images of TBX21 in CRC primary and liver metastatic tumor tissues. Scale bar: 50 μm. **I.** Quantification of TBX21 expression in CRC primary and liver metastatic tumor tissues. *p<0.05.

To investigate the expression pattern of TBX21 in CRC, we first conducted bioinformatics analyses using the TIMER and UALCAN databases. The results demonstrated that TBX21 expression in CRC tissues was considerably lower than in corresponding normal tissues across both databases (Figure 1A, B). To validate these findings, immunohistochemistry was performed on a human CRC tissue microarray containing 150 samples (50 normal and 100 cancer tissue samples) using a TBX21-specific antibody. Tumor samples exhibited lower TBX21 expression compared to normal tissues (Figure 1C, D). We further analyzed the relationship between TBX21 expression and TNM stage in tumor samples. The results indicated a negative correlation between high TNM stage in tumor samples and elevated TBX21 expression (Figure 1E, F). Additionally, western blot analysis was performed on 10 pairs of fresh CRC and adjacent non-tumor tissues to further validate these findings (Figure 1G, Figure S1A), revealing reduced TBX21 levels in CRC tissues and higher levels in adjacent non-tumor tissues. To examine the relationship between TBX21 expression and CRC metastasis, we performed immunohistochemistry on human CRC tissue microarrays containing 68 primary and liver metastatic tumor samples (34 primary tumor tissues and 34 liver metastatic tumor tissues). We found that CRC liver metastatic samples exhibited lower TBX21 expression than primary tumor tissues (Figure 1H, I). Collectively, these findings suggest that TBX21 expression is downregulated in human CRC tissues and that TBX21 may act as a potential tumor suppressor in CRC metastasis.

### Ectopic expression of TBX21 inhibits CRC cell migration *in vitro*

**Figure 2:**
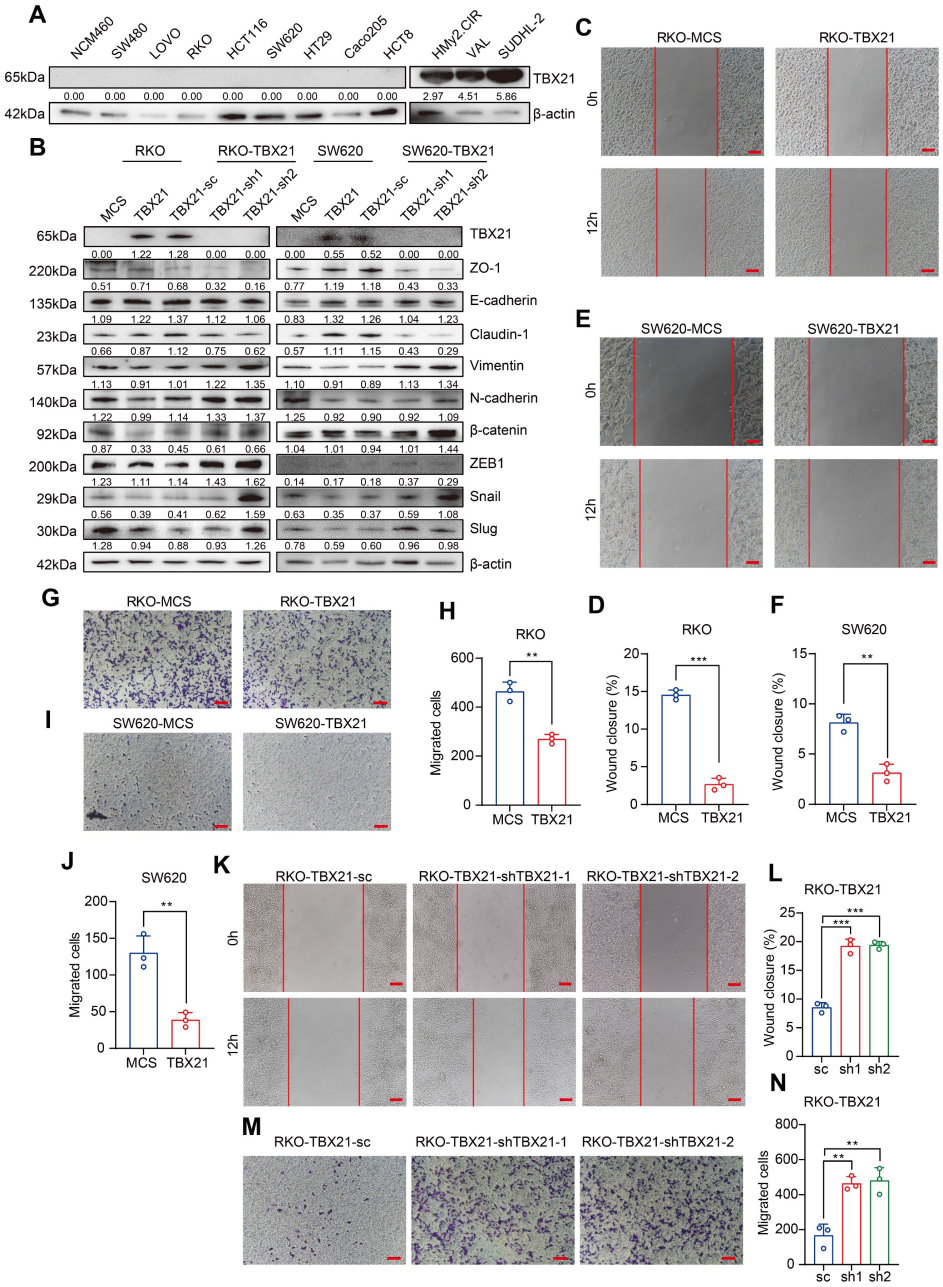
** Ectopic expression of TBX21 inhibits CRC cell migration *in vitro*. A.** TBX21 expression in normal and CRC cell lines analyzed by Western blotting, with the normal B cell line (HMy2.CIR) and DLBCL cell lines (VAL, SUDHL-2) as positive controls. **B.** Western blot analysis of TBX21 overexpression or knockdown efficiency and EMT marker expression in RKO and SW620 cell lines, with β-actin as the loading control. **C & D.** Representative images of the wound healing assay and quantitative analysis of wound closure in RKO-TBX21 or MCS cells. N=3, ***p<0.001. Scale bar: 50 μm. **E & F.** Representative images of the wound healing assay and quantitative analysis of wound closure in SW620-TBX21 or MCS cells. N=3, **p<0.01. Scale bar: 50 μm. **G & H.** Representative images of the transwell assay and quantitative analysis of migrated cells in RKO-TBX21 or MCS cells. N=3, **p<0.01. **I & J.** Representative images of the transwell assay and quantitative analysis of migrated cells in SW620-TBX21 or MCS cells. N=3, **p<0.01. **K & L.** Representative images of the wound healing assay and quantitative analysis of wound closure in RKO-TBX21-shTBX21 or scrambled control (sc) cells. N=3, ***p<0.001. Scale bar: 50 μm. **M & N.** Representative images of the transwell assay and quantitative analysis of migrated cells in RKO-TBX21-shTBX21 or sc cells. N=3, **p<0.01. All experiments were repeated three times, with representative data shown from one experiment.

To determine whether findings from tumor samples could be replicated in CRC cell lines, we used qPCR and western blotting to examine TBX21 expression levels in normal colon cells and CRC cell lines. Unexpectedly, the results showed that TBX21 expression was absent in both normal colon cells and CRC cell lines (Figure S1B, Figure 2A, left) but was highly expressed in B cells and B lymphoma cells (Figure 2A, right).

To investigate the functional role of TBX21 in CRC cell migration, we ectopically expressed Flag-TBX21 in RKO and SW620 cell lines. Western blot results confirmed that TBX21 was overexpressed in both cell lines (Figure 2B). In wound-healing assays, ectopic TBX21 expression was observed to inhibit the migratory ability of both cell lines (Figure 2C, D, E, F). Similar findings were noted in transwell assays, where TBX21 ectopic expression inhibited the migration ability of both cell lines (Figure 2G, H, I, J). Further, qPCR and western blotting were conducted to assess the expression of metastasis-associated EMT markers. Results showed that ectopic TBX21 expression increased the expression of epithelial markers (e.g., ZO-1, E-cadherin, and Claudin-1) and decreased the expression of mesenchymal markers (e.g., Vimentin, N-cadherin, β-catenin, ZEB1, Slug, and Snail) (Figure 2B, Figure S2B, C). Together, these results indicate that ectopic TBX21 expression inhibits CRC cell migration *in vitro*.

### Reconstituted silencing of TBX21 promotes cell migration *in vitro*

To further investigate the role of TBX21 in the inhibition of CRC cell migration, we reconstituted specific shRNAs to suppress TBX21 expression in RKO-TBX21 cells. As shown in Figure 2B, TBX21 shRNA effectively reduced TBX21 expression. Moreover, wound healing assay results indicated that knockdown of TBX21 expression promoted cell migration at the indicated time points (Figure 2K, L). Consistent results were observed in the transwell assay (Figure 2M, N), where TBX21 knockdown enhanced the migration of RKO-TBX21 cells. Additionally, qPCR and western blotting analysis of EMT markers demonstrated that TBX21 knockdown increased the expression of N-cadherin, Vimentin, Snail, β-catenin, ZEB1 and Slug, while decreasing the expression of E-cadherin, ZO-1, and Claudin-1 (Figure 2B, Figure S2D). Collectively, these results highlight the important role of TBX21 in regulating tumor cell migration.

### Ectopic expression of TBX21 inhibits tumor metastasis *in vivo*

**Figure 3:**
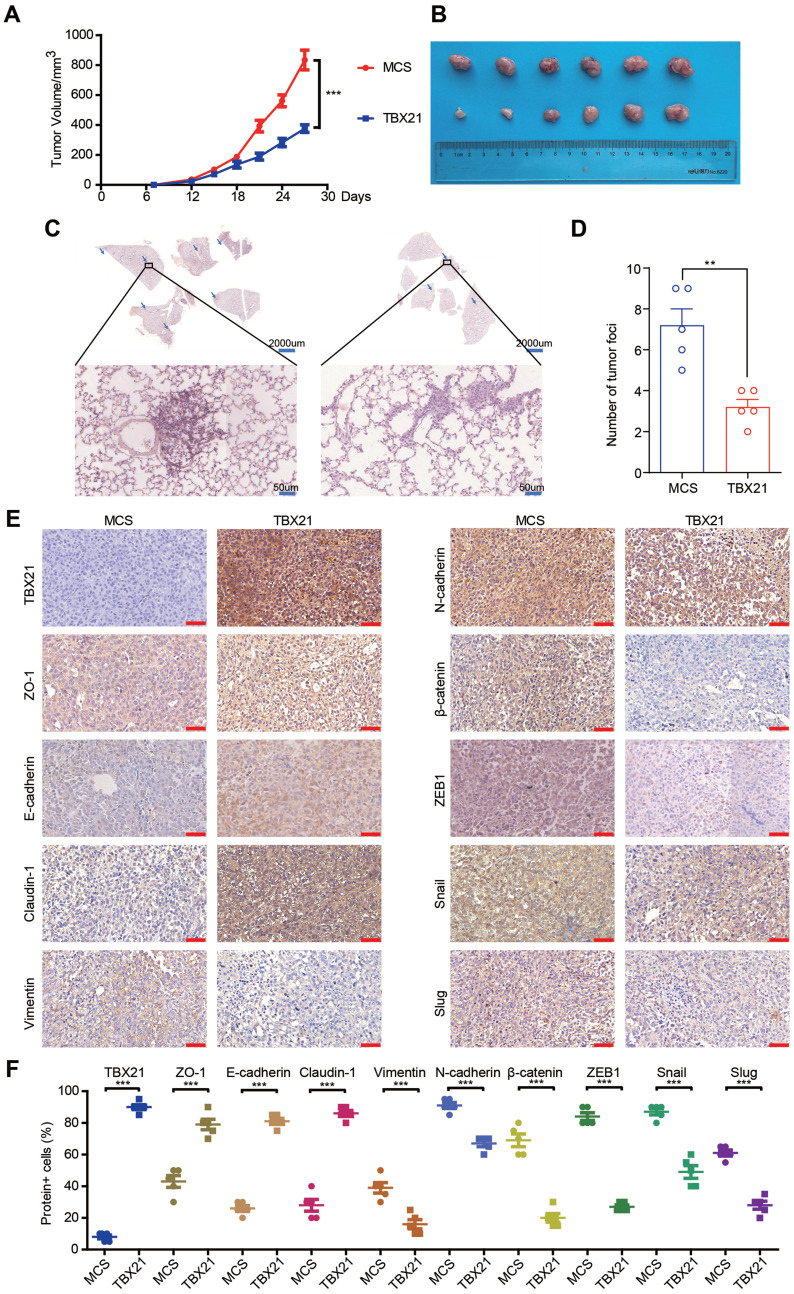
** Ectopic expression of TBX21 inhibits CRC growth and metastasis *in vivo*. A.** Stable RKO-MCS and RKO-TBX21 cells were injected into nude mice at the fourth fat pad, with tumor growth curves measured. N=6, ***p<0.001. **B.** Tumors isolated from the different groups of mice. **C & D.** H&E staining results and quantitative analysis of lung metastases in RKO-MCS and RKO-TBX21 groups. N=5, ***p<0.001. **E & F.** IHC staining for TBX21, ZO-1, E-cadherin, Claudin-1, Vimentin, N-cadherin, β-catenin, ZEB1, Slug, and Snail expression in tumor tissues, with quantitative analysis. N=5, ***p<0.001. The IHC experiment was repeated three times, with representative data shown from one experiment.

Since TBX21 inhibited the migratory properties of CRC cells *in vitro*, we explored its effects *in vivo* using a xenograft mouse model. For this purpose, the RKO-MCS and RKO-TBX21 stable cell lines were injected into the fourth fat pad of nude mice. As shown in Figure 3A, B, tumor growth and volume were markedly reduced in the RKO-TBX21 group compared to the control group. Moreover, HE staining of mouse lung tissue revealed a significant reduction in metastatic nodules and areas in the RKO-TBX21 group relative to the RKO-MCS group (Figure 3C, D). Additionally, IHC staining (Figure 3E, F) showed that ectopic TBX21 expression reduced the expression of Vimentin, N-cadherin, β-catenin, ZEB1, Slug, and Snail, and increased the expression of ZO-1, E-cadherin, and Claudin-1, consistent with *in vitro* findings. Collectively, these findings suggest that ectopic expression of TBX21 inhibits CRC metastasis *in vivo*.

### TBX21 inhibits CRC migration via suppression of GSK3β phosphorylation

**Figure 4:**
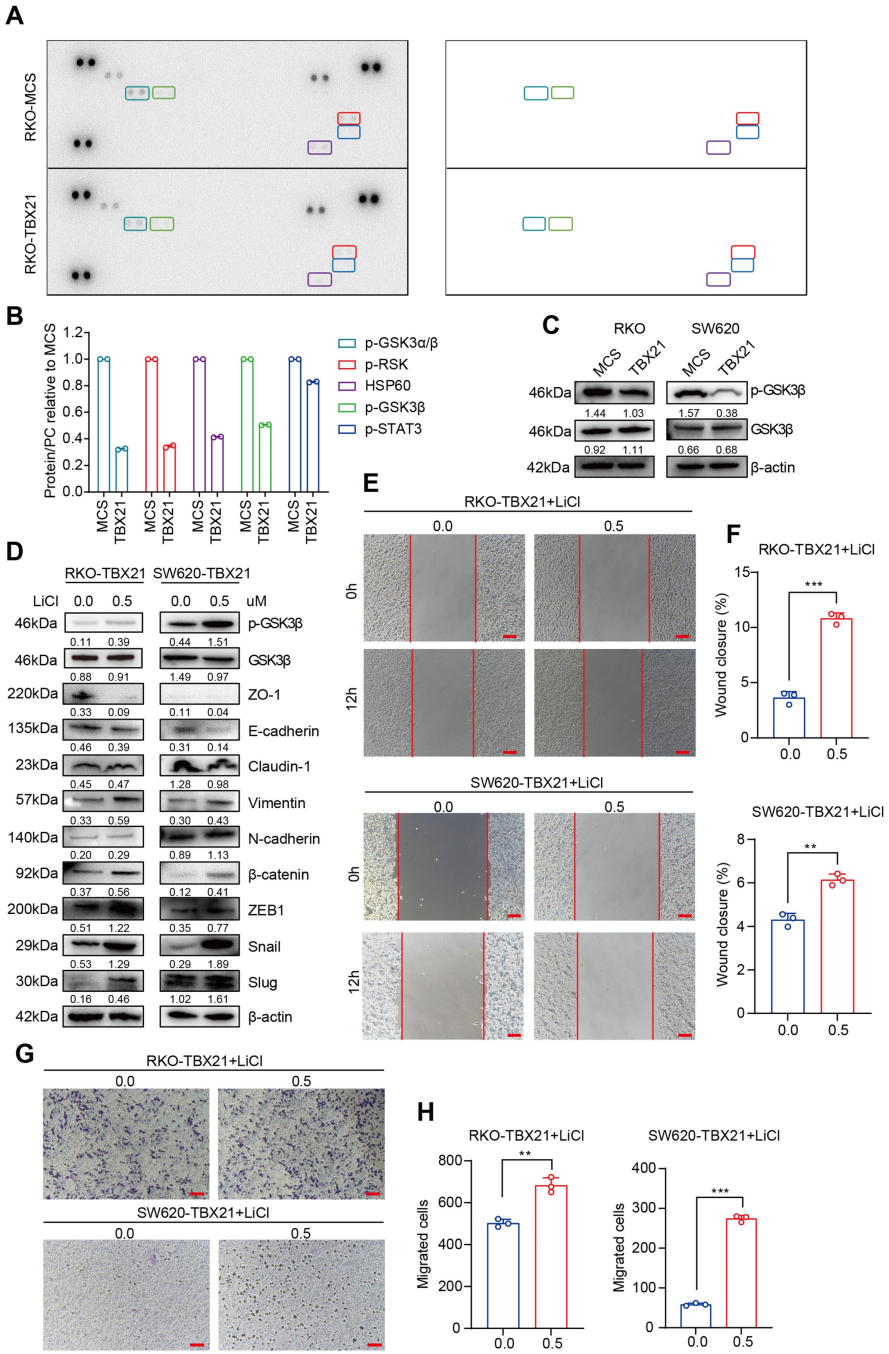
** TBX21 inhibits CRC migration by suppressing GSK3β phosphorylation. A & B.** Human phosphokinase arrays were used to detect activation of 39 kinases in RKO cells expressing TBX21 or MCS. **C.** Western blot analysis of GSK3β and p-GSK3β, with β-actin as the loading control. **D.** Western blot analysis of p-GSK3β, GSK3β, ZO-1, E-cadherin, Claudin-1, Vimentin, N-cadherin, β-catenin, ZEB1, Slug, and Snail expression in RKO-TBX21 and SW620-TBX21 cells with or without treated with 0.5 μM LiCl, with β-actin as the loading control. **E & F.** Representative wound healing assay images and quantitative analysis of wound closure in RKO-TBX21, SW620-TBX21 cells with or without treated with 0.5 μM LiCl. N=3, **p<0.01, ***p<0.001. Scale bar: 50 μm. **G & H.** Representative transwell assay images and quantitative analysis of migrated cells in RKO-TBX21, SW620-TBX21 cells with or without treated with 0.5 μM LiCl. N=3, **p<0.01, ***p<0.001. Experiments (C-H) were repeated three times, with representative data shown from one experiment.

To elucidate the potential mechanism through which TBX21 inhibits CRC cell migration, we conducted a protein kinase microarray analysis on RKO-MCS and RKO-TBX21 cells, assessing 37 kinase phosphorylation profiles. As shown in Figure 4A, B, ectopic expression of TBX21 reduced the expression of multiple phosphokinases, particularly p-GSK3β. To confirm these results, we performed western blotting to evaluate GSK3β phosphorylation in RKO and SW620 cell lines, showing that ectopic expression of TBX21 decreased p-GSK3β levels in both cell types (Figure 4C).

To assess the role of GSK3β phosphorylation in TBX21-mediated inhibition of CRC migration, the GSK3β phosphorylation activator LiCl was used to increase GSK3β phosphorylation. As shown in Figure 4D, treatment with 0.5 µM LiCl effectively promoted GSK3β phosphorylation in RKO-TBX21 and SW620-TBX21 cells. The wound healing (Figure 4E, F) and transwell (Figure 4G, H) assays demonstrated that increased p-GSK3β reversed the TBX21-mediated inhibition of CRC cell migration.

To determine whether the LiCl-induced changes in migration were dependent on TBX21 expression, we treated RKO-MCS and SW620-MCS cells with LiCl. Results from the wound healing (Figure S2A, B, C, D) and transwell (Figure S2E, F, G, H) assays showed that LiCl treatment significantly enhanced cell migration independently of TBX21 expression.

Overall, these findings suggest that TBX21 suppresses GSK3β phosphorylation and relies on this suppression to inhibit CRC cell migration.

### Analysis and identification of TBX21 downstream relevant target genes

**Figure 5:**
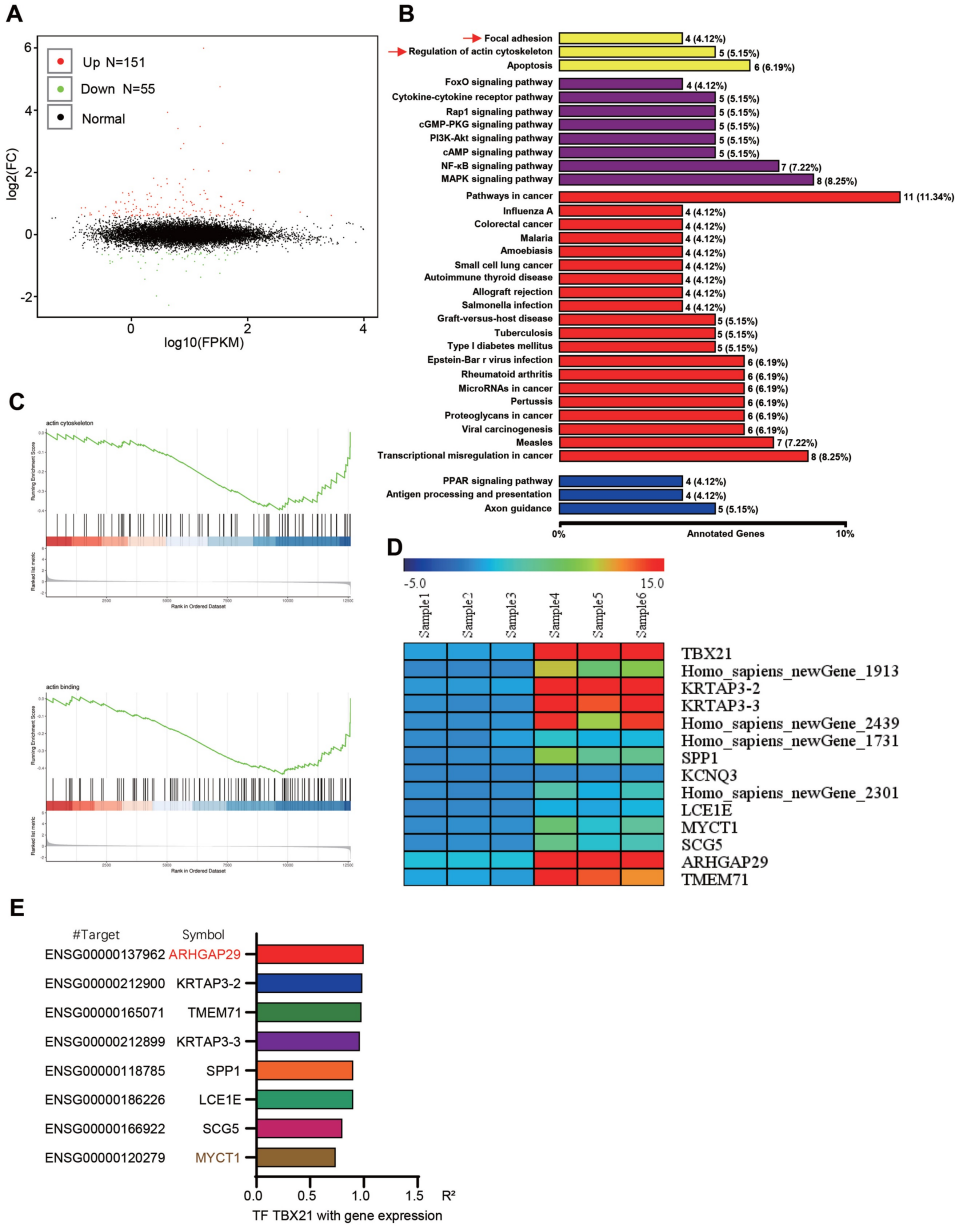
**Analysis and identification of TBX21 downstream target genes. A.** Volcano plot of RNA-seq results. **B.** KEGG pathway analysis of RNA-seq results. **C.** GSEA of RNA-seq results. **D.** Ten genes with the most significant expression differences. **E.** Transcription factor-target gene activity analysis.

To further explore the potential mechanism underlying TBX21-mediated inhibition of GSK3β phosphorylation, RNA sequencing analysis was conducted, leading to the identification of new TBX21 target genes. The volcano plot results showed that ectopic expression of TBX21 upregulated 151 DEGs and downregulated 55 DEGs (Figure 5A). GO results indicated that TBX21 expression impacted cellular processes, such as focal adhesion and actin cytoskeleton regulation (Figure 5B). Consistently, GSEA results showed that TBX21 expression negatively regulated the actin backbone and actin binding (Figure 5C). To identify new TBX21 targets, we selected 14 genes with the most significant changes following TBX21 expression, including four unnamed genes (Figure 5D). Integrating this data with transcription factor target gene activity analysis, we identified ARHGAP29 and MYCT1 as potential target genes (Figure 5E). In conclusion, our analysis identified downstream genes functionally relevant to TBX21 activity.

### TBX21 directly regulates ARHGAP29 expression to mediate GSK3β phosphorylation and inhibit cell migration

**Figure 6:**
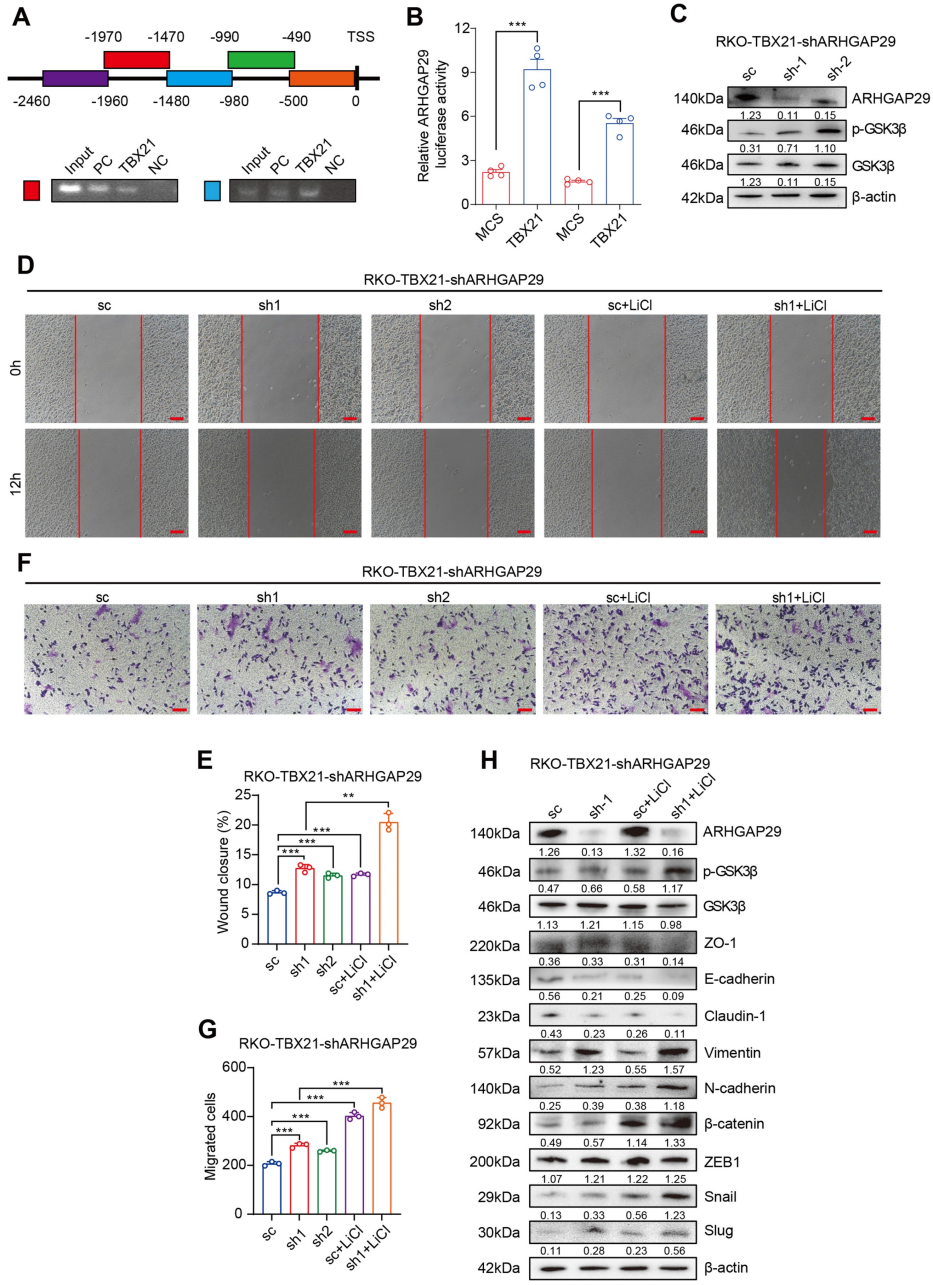
** TBX21 directly regulates ARHGAP29 expression, mediating GSK3β phosphorylation and cell migration inhibition. A.** ChIP assay of TBX21 binding sites on the ARHGAP29 promoter in RKO-TBX21 cells; PC indicates positive control (H3K27 antibody), and NC indicates negative control (IgG). **B.** Dual-luciferase assay of ARHGAP29 promoter activity in RKO and SW620 cells with ectopic TBX21 expression or MCS. N=3, ***p<0.001. **C.** Western blot analysis of ARHGAP29 knockdown efficiency and p-GSK3β expression in RKO-TBX21-shARHGAP29 or sc cells. **D-G.** Representative wound healing and transwell assay images and quantitative analysis for RKO-TBX21 sc and shARHGAP29 cells with or without 0.5 μM LiCl. N=3, **p<0.01, ***p<0.001. Scale bar: 50 μm. **H.** Western blot analysis of ZO-1, E-cadherin, Claudin-1, Vimentin, N-cadherin, β-catenin, ZEB1, Slug, and Snail in RKO-TBX21-shARHGAP29 or sc cells, with β-actin as the loading control. Experiments were repeated three times, with representative data shown from one experiment.

Previously, we demonstrated that TBX21 inhibits RSK and GSK3β activation by upregulating ARHGAP29, which in turn affects CRC cell proliferation and apoptosis. To validate the regulatory role of TBX21 on ARHGAP29, we performed chromatin immunoprecipitation (ChIP)-PCR (Figure 6A) and dual-luciferase reporter assays (Figure 6B). The results indicated that TBX21 binds to the -980bp to -1970bp region of the ARHGAP29 promoter to positively regulate ARHGAP29 expression.

To investigate the role of ARHGAP29 in TBX21-mediated inhibition of CRC cell migration, we used a specific shRNA to knock down ARHGAP29 expression in RKO-TBX21 cells. Western blot analysis confirmed that the ARHGAP29 shRNA effectively inhibited ARHGAP29 expression in RKO-TBX21 cells (Figure 6C). We also observed that ARHGAP29 knockdown increased the level of p-GSK3β, while the total GSK3β level remained almost unchanged (Figure 6C). Additionally, wound healing assays showed that ARHGAP29 knockdown reversed the TBX21-mediated inhibition of cell migration (Figure 6D, E). Similar results were seen in transwell assays, where ARHGAP29 knockdown eliminated the TBX21-mediated reduction in cell migration (Figure 6F, G). Furthermore, ARHGAP29 knockdown in RKO-TBX21 cells reduced epithelial cell marker expression (ZO-1, E-cadherin, and Claudin-1) while increasing mesenchymal marker expression (Vimentin, N-cadherin, β-catenin, ZEB1, Slug, and Snail) (Figure 6H).

To assess the combined effect of ARHGAP29 knockdown and lithium chloride (LiCl) treatment on TBX21-mediated GSK3β phosphorylation, we treated ARHGAP29-knockdown cells with LiCl. Western blot analysis revealed a marked increase in p-GSK3β and mesenchymal marker expression, along with a reduction in epithelial markers (Figure 6H). Moreover, wound healing and transwell assays demonstrated that ARHGAP29 knockdown, combined with LiCl treatment, significantly enhanced cell migration and eliminated TBX21-mediated migration inhibition (Figure 6D, E, F, G).

To determine whether the effects of ARHGAP29 on p-GSK3β and cell migration depend on TBX21 expression, we knocked down ARHGAP29 in RKO-MCS cells and treated them with LiCl. Western blotting confirmed efficient ARHGAP29 knockdown (Figure S3A). Wound healing and transwell assays revealed that ARHGAP29 knockdown promoted cell migration, and this effect was further enhanced with LiCl treatment (Figure S3B, C, D, E). Additionally, western blot analysis of EMT markers showed increased mesenchymal and reduced epithelial marker expression following ARHGAP29 knockdown and ARHGAP29 knockdown combined with LiCl treatment (Figure S3F). These effects were independent of TBX21 expression.

Thus, we conclude that TBX21 directly regulates ARHGAP29 expression to mediate GSK3β phosphorylation and cell migration inhibition.

### MYCT1 interacts with ZO-1 to regulate the cytoskeleton, essential for TBX21-mediated migration inhibition

**Figure 7:**
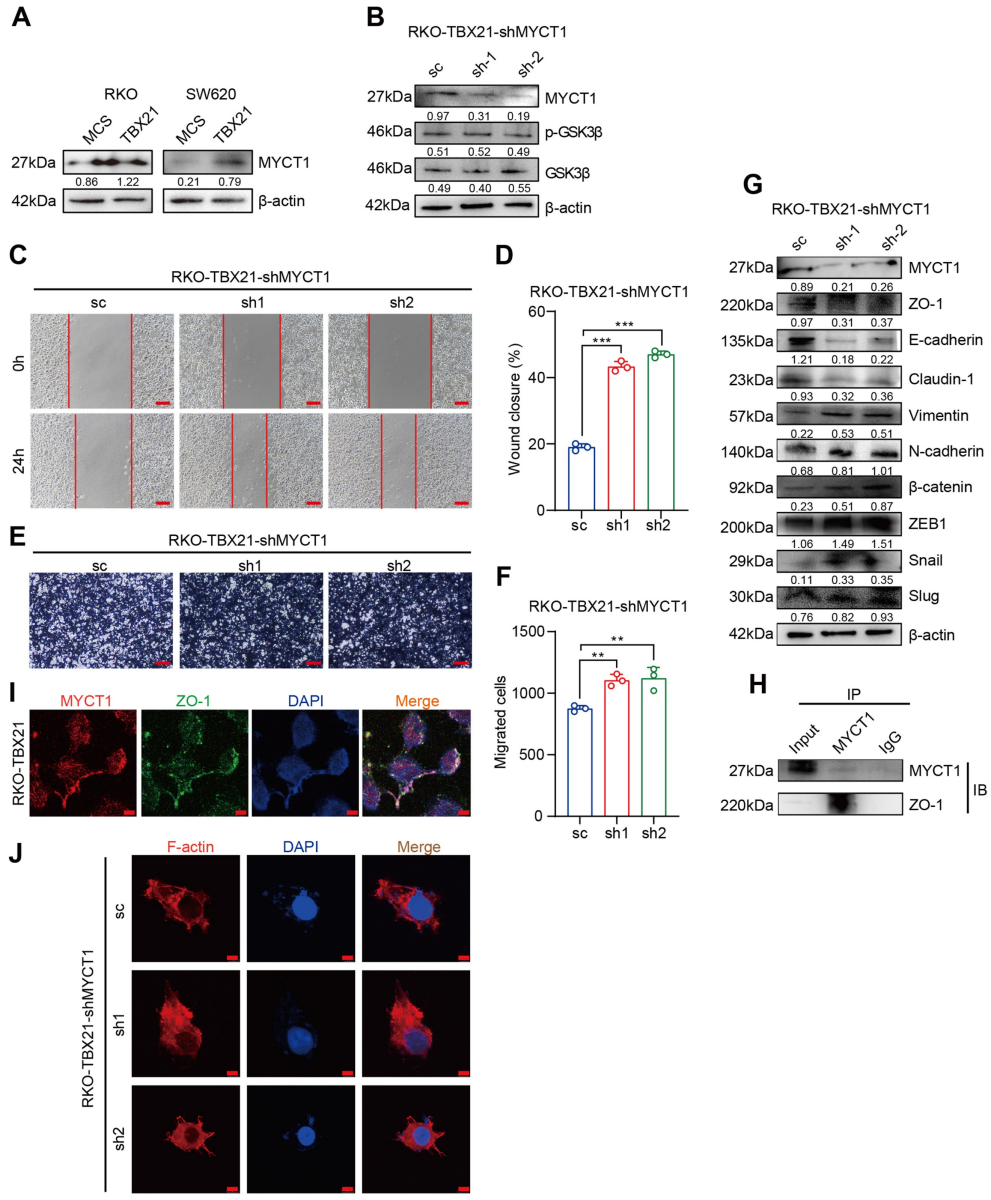
** MYCT1 interaction with ZO-1 in cytoskeleton regulation is critical for TBX21-mediated migration inhibition. A-B.** Western blot analysis of MYCT1 expression in RKO and SW620 cells overexpressing TBX21 and MYCT1 knockdown efficiency with p-GSK3β expression in RKO-TBX21-shMYCT1 or sc cells. **C-F.** Representative wound healing and transwell assay images and quantitative analysis of RKO-TBX21-shMYCT1 and sc cells. N=3, **p<0.01, ***p<0.001. Scale bar: 50 μm. **G.** Western blot analysis of ZO-1, E-cadherin, Claudin-1, Vimentin, N-cadherin, β-catenin, ZEB1, Slug, and Snail in RKO-TBX21-shMYCT1 and sc cells, with β-actin as the loading control. **H.** Immunoprecipitation (IP) assay for MYCT1 and ZO-1 binding. **I.** Immunofluorescence assay of MYCT1 and ZO-1 co-localization. Scale bar: 20 μm. **J.** Actin-Tracker Red-Rhodamine staining for F-actin in RKO-TBX21-shMYCT1 and sc cells. Scale bar: 20 μm. All experiments were repeated three times, with representative data shown from one experiment.

Among the candidate target genes, MYCT1 showed a strong association with tumor cell migration. We validated the RNA sequencing results by western blotting, confirming that MYCT1 expression increased following TBX21 overexpression in RKO and SW620 cells (Figure 7A). To investigate the role of MYCT1 in TBX21-mediated inhibition of CRC cell migration, we used specific shRNAs to knock down MYCT1. As shown in Figure 7B, MYCT1 was efficiently knocked down in RKO-TBX21 cells. Wound healing assays demonstrated that MYCT1 knockdown reversed the TBX21-mediated inhibition of cell migration (Figure 7C, D). Similarly, transwell assays showed that MYCT1 knockdown abolished the TBX21-mediated decrease in cell migration (Figure 7E, F). Moreover, MYCT1 knockdown in RKO-TBX21 cells reduced epithelial marker expression (ZO-1, E-cadherin, and Claudin-1) while increasing mesenchymal marker expression (Vimentin, N-cadherin, β-catenin, ZEB1, Slug and Snail) (Figure 7G).

Interestingly, MYCT1 knockdown did not affect GSK3β phosphorylation (Figure 7B), suggesting that MYCT1 may modulate cell migration through a GSK3β-independent pathway. MYCT1 interacts with ZO-1 to regulate the cytoskeleton. To further explore MYCT1's role in TBX21-mediated inhibition of CRC metastasis, we performed immunoprecipitation assays, revealing that MYCT1 interacts with ZO-1 (Figure 7H). Immunofluorescence analysis showed co-localization of MYCT1 and ZO-1, consistent with the findings above (Figure 7I). Additionally, actin-tracker red-rhodamine staining of F-actin revealed weakened cell polarity following MYCT1 knockdown, indicating enhanced migratory ability (Figure 7J). Overall, these findings suggest that MYCT1 interaction with ZO-1 to regulate the cytoskeleton is essential for TBX21-mediated inhibition of cell migration.

### Combined MYCT1-shRNA and LiCl treatment reduces TBX21-mediated tumor metastasis and progression inhibition *in vivo*

**Figure 8:**
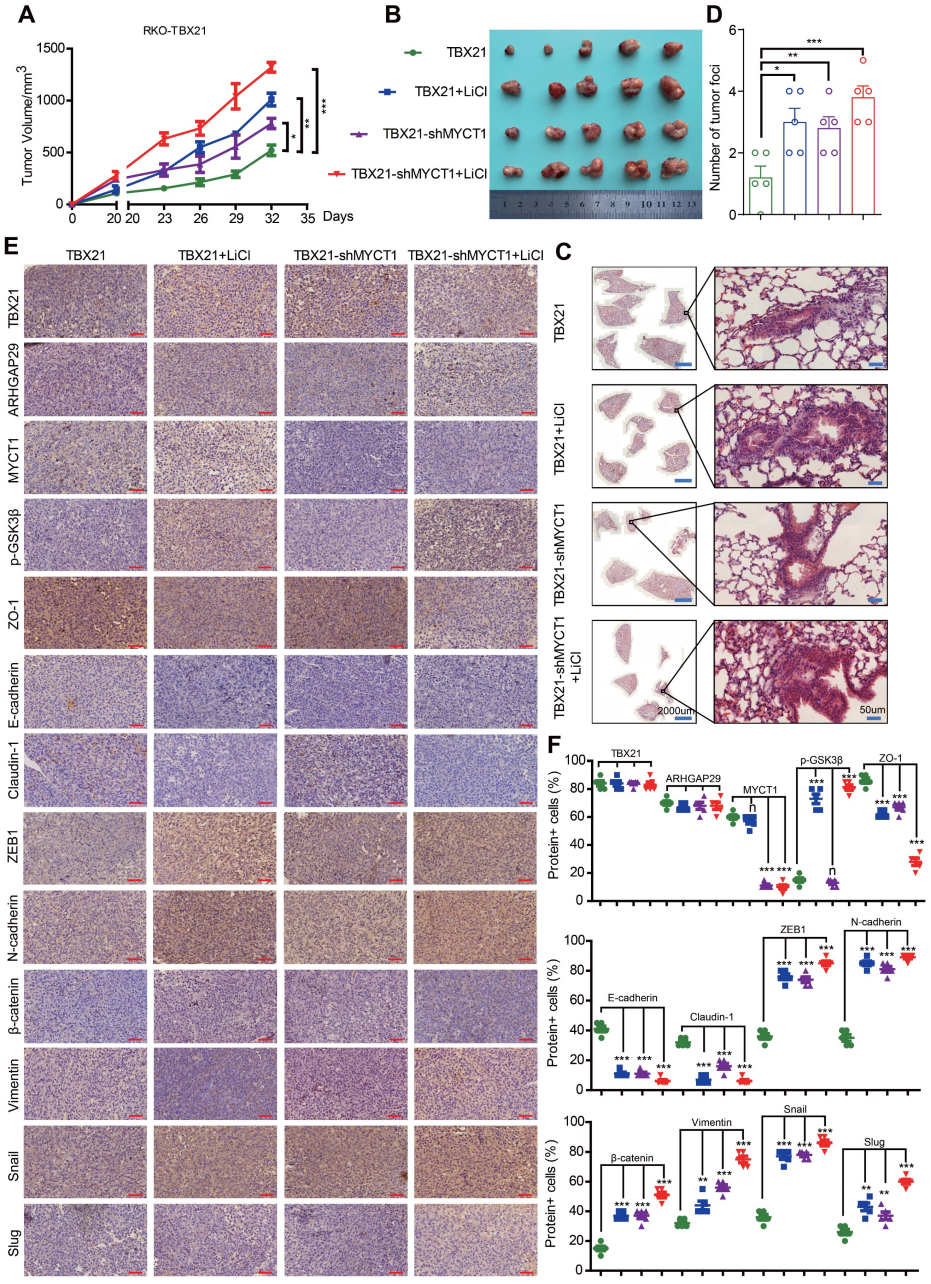
** Combined MYCT1-shRNA and LiCl treatment diminished TBX21-mediated tumor metastasis and progression inhibition *in vivo*. A.** Nude mice were injected with stable RKO-TBX21-sc or RKO-TBX21-shMYCT1 cells into the fourth fat pad and then treated or not treated with LiCl. Tumor growth curves were measured. N = 5, *p < 0.05, **p < 0.01, ***p < 0.001. **B.** Tumors separated from mice in different groups are shown. **C.** Representative images of H&E staining of lungs from different groups are displayed. Left, scale bar: 2000 μm, right, scale bar: 50 μm. **D.** Statistical results of metastatic nodules in the lungs from various groups are shown. N = 5, *p < 0.05, **p < 0.01, ***p < 0.001. **E.** Immunohistochemical (IHC) staining assays were conducted to assess the expression of TBX21, ARHGAP29, MYCT1, p-GSK3β, and epithelial-mesenchymal transition (EMT) markers in tumors from various groups of mice. Scale bar: 50 μm. **F.** The statistical results of positive cells for TBX21, ARHGAP29, MYCT1, p-GSK3β, and EMT markers in tumors from various groups of mice are presented. N = 5, n means none sense, **p < 0.01, ***p < 0.001. The H&E and IHC experiments were repeated three times, and data are displayed once.

We then investigated the effects of combination treatment with MYCT1-shRNA and the GSK3β phosphorylation agonist LiCl on TBX21-mediated inhibition of CRC metastasis in a xenograft mouse model *in vivo*. Stable RKO-TBX21 or RKO-TBX21-shMYCT1 cells were injected into the fourth mammary fat pad of nude mice and treated with LiCl for 20 days. As shown in Figure 8A, B, the combination of RKO-TBX21-shMYCT1 and LiCl was more effective in abolishing TBX21-mediated inhibition of tumor growth and volume than either treatment alone. HE staining of mouse lungs showed an increase in metastatic nodules and areas in the shMYCT1, LiCl, and shMYCT1 + LiCl groups relative to the control (Figure 8C, D). IHC results indicated that epithelial marker expression was significantly reduced, while mesenchymal marker expression increased significantly in the treatment groups compared to the control (Figure 8E, F).

To verify *in vivo* whether the effects of MYCT1 and LiCl treatment on CRC metastasis were dependent on TBX21 expression, we first generated a cell line with stable knockdown of MYCT1 expression using RKO-MCS cells and confirmed the knockdown efficiency (Figure S4A). We then injected either the RKO-MCS or RKO-MCS-shMYCT1 stable cell lines into the fourth mammary fat pad of nude mice and treated them with LiCl 20 days post-injection. As shown in Figure S4B, C, knockdown of MYCT1 and treatment with LiCl independently inhibited tumor growth and volume, while the combination of MYCT1 knockdown with LiCl was more effective than either treatment alone. HE staining of mouse lungs showed an increase in metastatic nodules and areas in the shMYCT1, LiCl, and shMYCT1 + LiCl groups compared to the control group (Figure S4D). These effects were independent of TBX21 expression.

These findings suggest that MYCT1-shRNA and LiCl treatment reduce TBX21-mediated inhibition of tumor metastasis and progression *in vivo*.

### Proposed model of TBX21 in inhibiting CRC metastasis

**Figure 9:**
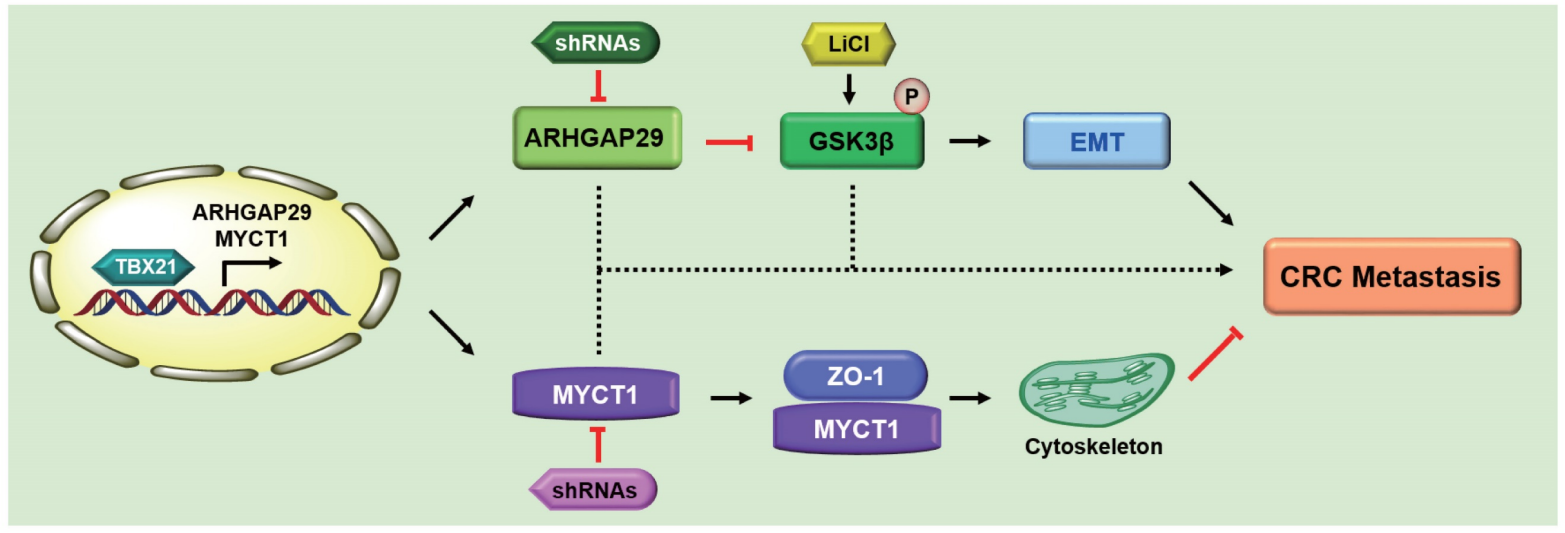
Proposed model of TBX21 in inhibiting CRC metastasis.

Based on these findings, we propose the following model (Figure 9): As a nuclear transcription factor, TBX21 directly binds the promoter of ARHGAP29, promoting ARHGAP29 expression, which in turn inhibits GSK3β phosphorylation, affecting the EMT process. TBX21 promotes MYCT1 expression, which interacts with ZO-1 to regulate the cytoskeleton. Together, ARHGAP29/GSK3β and MYCT1/ZO-1 synergistically suppress CRC cell metastasis. ShRNAs targeting ARHGAP29 or MYCT1, as well as LiCl, which promotes GSK3β phosphorylation, were able to abolish TBX21-mediated CRC cell metastasis inhibition *in vitro* and *in vivo*.

## Discussion

Colorectal cancer (CRC) is one of the most prevalent and fatal cancers worldwide. Approximately 20% of CRC patients have metastases at the time of diagnosis 37. Metastatic colorectal cancer has a low likelihood of requiring surgery and lacks appropriate treatment 5. The discovery of early diagnostic markers and therapeutic targets for colorectal cancer may provide new strategies for the treatment of metastatic colorectal cancer.

TBX21 is an immune cell-specific member of the T-box family of transcription factors that coordinates the differentiation and function of various immune cells. Studies have shown that TBX21 activates the expression of the Th1 signature cytokine IFN-γ and inhibits IL-4 production in developing Th2 cells 38. T-bet expression in dendritic cells (DCs) is necessary for the initiation of antigen-specific CD4^+^ T cells 39. TBX21 is the only gene with altered expression and methylation associated with CTL infiltration 40. In the absence of T-bet, CD8^+^ T cells produce less IFN-γ and exhibit reduced cytolytic activity 41; B cells do not produce immunoglobulin G2a (IgG2a) 42; and natural killer (NK) and NKT cells fail to develop properly and become dysfunctional 43. TBX21 coordinates multiple aspects of the immune response and has been implicated in the development of systemic multisystem diseases.

Research has shown that during progressive obesity, T-bet^+^ B cells accumulate in adipose tissue and drive the production of IgG2c and CXCL10, exacerbating metabolic disorders and pancreatic damage 44. An increased frequency of CD11c^+^Tbet^+^CD211low B cells is a hallmark of immune dysregulation in Down syndrome. Several single nucleotide polymorphisms of TBX21 are associated with lupus erythematosus, autoimmune hepatitis type 1, major depressive disorder, periapical inflammation 45, and asthma 12, 46. With advances in tumor immunity research, antitumor immune responses are increasingly recognized as critical to tumor development. As an immune-related molecule, TBX21 has received attention for its role in tumors. The current study found that TBX21 plays a key role in lung carcinogenesis via the TBX21/IL-4 signaling pathway 14. TBX21 inhibits the proliferation of colon cancer cells through an ARHGAP29/RSK/GSK3β-dependent mechanism 15. Additionally, TBX21 methylation is a potential regulator of immunosuppression in CMS1 subtype CRC 40. However, the role of TBX21 in metastasis remains unclear. Our results suggest that TBX21 may function as a tumor suppressor in colorectal cancer, with its overexpression inhibiting CRC cell migration and EMT. Mechanistic studies suggest that TBX21 inhibits GSK3β activation by regulating the expression of the target gene ARHGAP29. Additionally, TBX21 influences the cytoskeleton and cell polarity by controlling the interaction between MYCT1 and ZO-1, thereby inhibiting CRC metastasis.

ARHGAP29 is a RhoGAP for RhoA and a target gene of the YAP/TAZ-TEAD transcription factor complex 17. Recent studies have shown that ARHGAP29 is a direct target gene of TBX21, is positively regulated by TBX21, and plays an important role in inhibiting the proliferation and promoting apoptosis of colon cancer cells 15. Several studies have shown that ARHGAP29 expression is closely associated with cell migration. Actin filament-binding protein (afadin) promotes vascular endothelial cell network formation and migration induced by vascular endothelial growth factor through inactivation of Rho-associated kinases via ARHGAP29 47. ARHGAP29 is required for keratinocyte morphology, proliferation, and migration mediated by the RhoA pathway. RhoA GTPase plays a key role in actin cytoskeleton remodeling, which is essential for controlling a variety of cellular functions, including cell proliferation, adhesion, migration, and shape changes. Furthermore, YAP regulates the destabilization of cytoskeleton-associated F-actin via ARHGAP29 48. In gastric cancer, YAP promotes ARHGAP29 expression, which, in turn, inhibits the RhoA-LIMK-cofilin pathway and destabilizes F-actin, thereby promoting gastric cancer cell migration 49. In colon cancer, LKB1 deficiency promotes CRC cell metastasis through TNIK upregulation and cytoskeletal remodeling mediated by the interaction between TNIK and ARHGAP29 50. circTMEM181 inhibits hepatocellular carcinoma migration and invasion by sponging miR-519a-5p and upregulating ARHGAP29 expression 18. In this study, using protein kinase microarray experiments, we revealed a novel molecular mechanism by which TBX21 inhibits CRC metastasis. ARHGAP29, as a direct target gene of TBX21, inhibited GSK3β activation, suppressed the EMT process, and thereby inhibited CRC metastasis. However, further investigation is needed to elucidate the specific molecular mechanism and role of cytoskeletal remodeling by ARHGAP29 in colon cancer metastasis to identify new therapeutic targets for colorectal cancer.

MYCT1, a novel c-Myc target gene, is regulated by multiple transcription factors. Studies have shown that MYCT1 is aberrantly expressed in various cancers and affects key biological processes such as proliferation, apoptosis, migration, genomic instability, and differentiation in cancer cells 25. Several studies have demonstrated that MYCT1 is associated with tumor cell migration. MYCT1 inhibits EMT and migration in laryngeal cancer cells 28, 51. MYCT1 plays a role in the growth and invasion of hepatocellular carcinoma cells 31 and is associated with immune infiltration in lung squamous cell carcinoma 52. Additionally, MYCT1 interacts with the tight junction protein ZO-1 to regulate Rho GTPase-mediated actin cytoskeleton dynamics 53, thereby promoting endothelial motility in angiogenic settings. Our study revealed that TBX21 acts on its target gene MYCT1, which interacts with ZO-1 to regulate the cytoskeleton and inhibit CRC migration. Targeting MYCT1 for vascular control in conjunction with immunotherapy may offer an effective therapeutic strategy.

In conclusion, we demonstrated that TBX21 expression was markedly downregulated in CRC tissues and was negatively correlated with the TNM stage of CRC. We found that ectopic expression of TBX21 inhibited CRC cell migration *in vitro* and metastasis *in vivo*. Additionally, we revealed that TBX21 directly binds to the promoter region of ARHGAP29, promoting ARHGAP29 expression, which in turn inhibits GSK3β phosphorylation and suppresses CRC metastasis. Furthermore, we showed that TBX21 promotes expression of the target gene MYCT1, and MYCT1 interacts with ZO-1 to regulate the cytoskeleton, thereby inhibiting CRC metastasis. Together, ARHGAP29/GSK3β and MYCT1/ZO-1 pathways synergistically suppress CRC cell metastasis. Our findings suggest that TBX21 could serve as a novel therapeutic target for metastatic CRC.

## Supplementary Material

Supplementary figures and tables.
