# Supplementary material for: TBX21 inhibits colorectal cancer metastasis through ARHGAP29/GSK3β inhibitory signaling- and MYCT1/ZO-1 signaling-dependent manner
Source: Int J Biol Sci. 2025 Jan 1;21(1):328–45. doi: 10.7150/ijbs.97920 (PMC11667819; doi:10.7150/ijbs.97920)
Supplement: Supplementary file 1 — Supplementary figures and tables. [file ijbsv21p0328s1.pdf]

Supplementary Figures and Materials

TBX21 inhibits colorectal cancer metastasis through ARHGAP29/GSK3β inhibitory signaling- and MYCT1/ZO-1 signaling-dependent manner

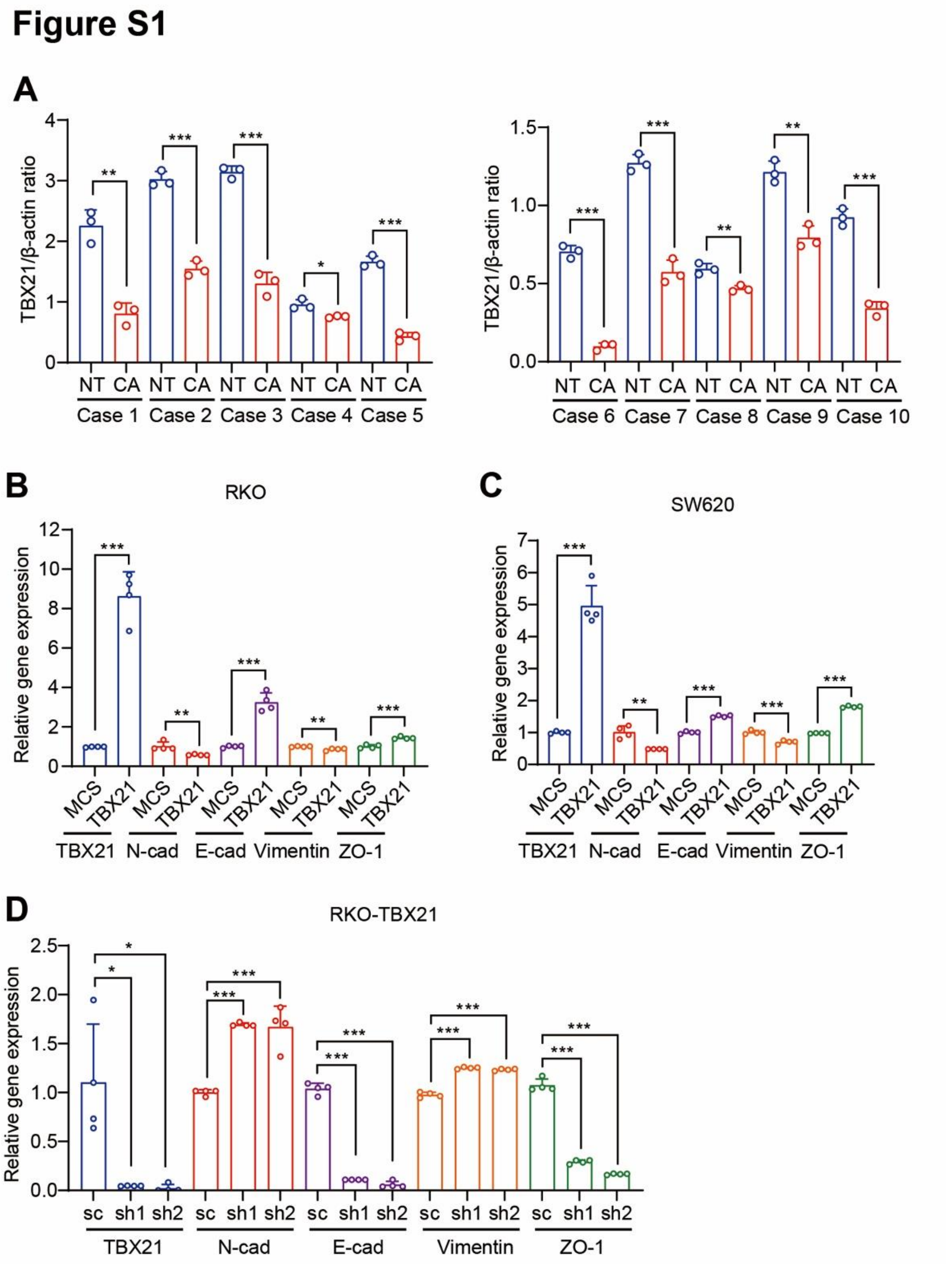

Figure S1. Western blot quantitative results and qPCR results. A. Quantitative results of figure 1G. **B.** QPCR was performed to check the mRNA expression of TBX21, N-cad, E-cad, Vimentin and ZO-1 in RKO-MCS and RKO-TBX21 cells. **C.** QPCR was performed to check the mRNA expression of TBX21, N-cad, E-cad, Vimentin and ZO-1 in SW620-MCS and SW620-TBX21 cells. **D.** QPCR was performed to check the mRNA expression of TBX21, N-cad, E-cad, Vimentin and ZO-1 in RKO-TBX21-sc, RKO-TBX21-shTBX21-1 and RKO-TBX21-shTBX21-2 cells.

Figure S2

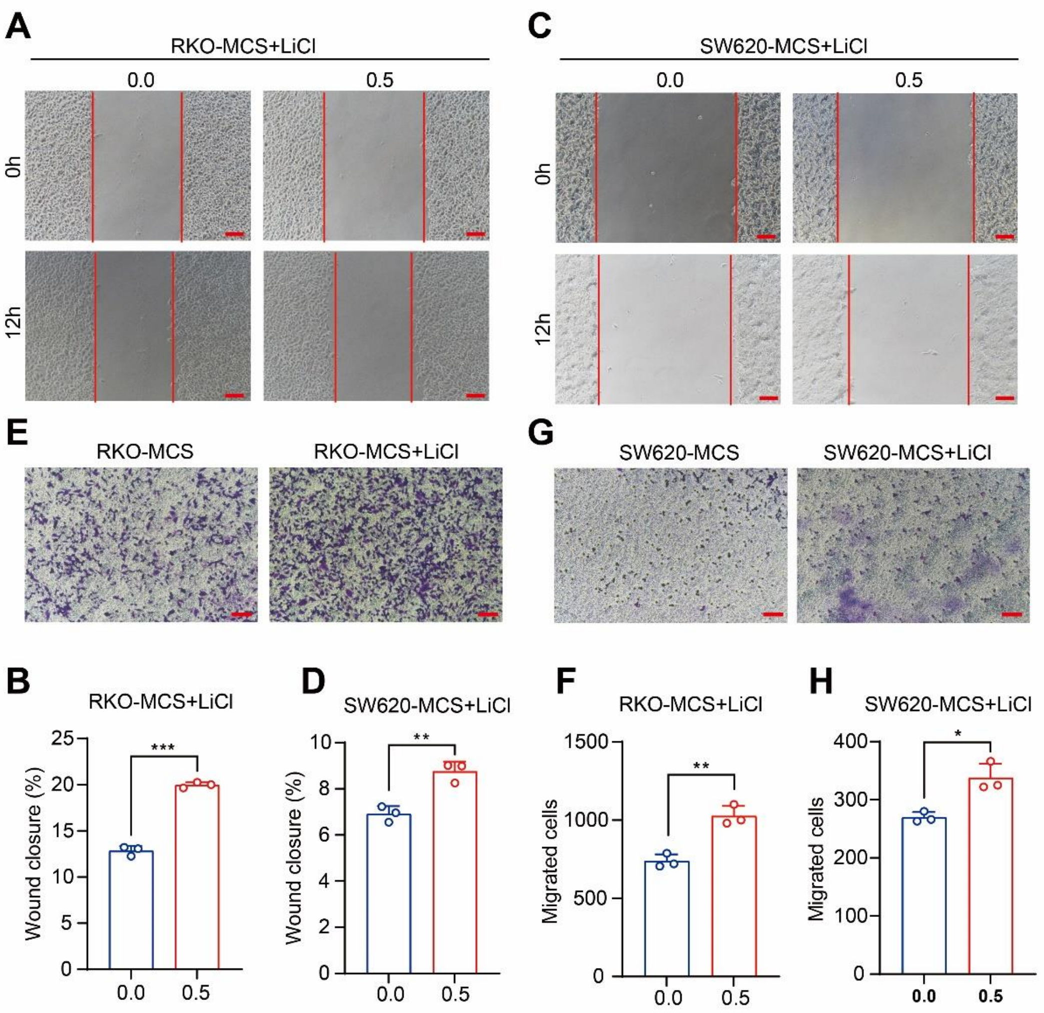

Figure S2. LiCl treatment promotes CRC cell migration *in vitro*. A & B. Representative images of wound healing assay and statistic results on cell wound closure in RKO-MCS cells treated with or without LiCl (0.5 μM) were shown. N=3, ***p<0.001. Scale bar: 50 μm. **C & D.** Representative images of wound healing assay and statistic results on cell wound closure in SW620-MCS cells treated with or without LiCl (0.5 μM) were shown. N=3, **p<0.01. Scale bar: 50 μm. **E & F.** Representative images of trans-well assay and statistic results on migrated cells in RKO-MCS cells treated with or without LiCl (0.5 μM) were shown. N=3, **p<0.01. Scale bar: 50 μm. **G & H.** Representative images of trans-well assay and statistic results on migrated cells in SW620-MCS cells treated with or without LiCl (0.5 μM) were shown. N=3, *p<0.05. Scale bar: 50 μm.

Figure S3

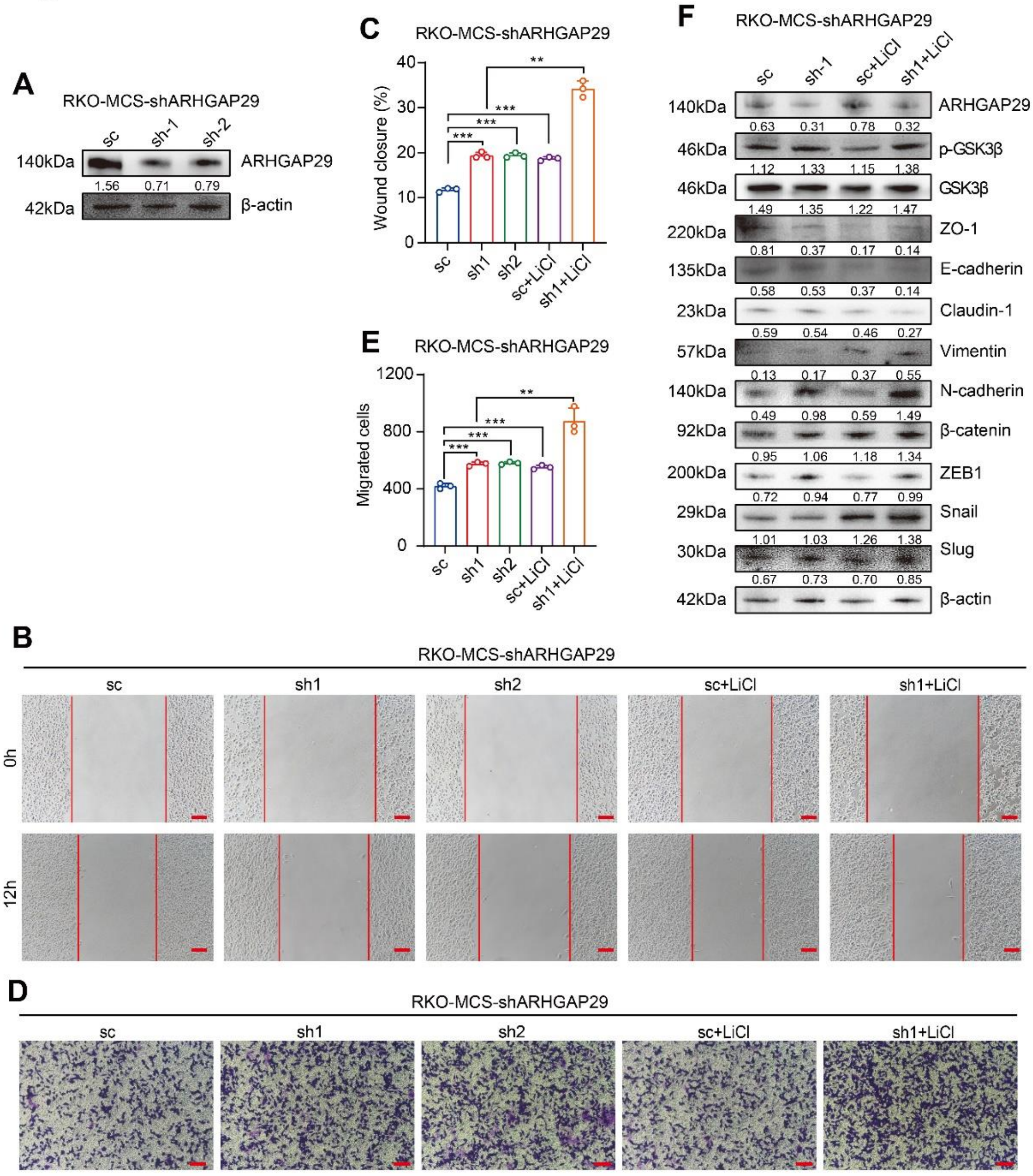

Figure S3. Knockdown ARHGAP29 promotes CRC cell migration *in vitro*. A. Western blot to analysis ARHGAP29 knockdown efficiency in RKO-MCS cells. **B & C.** Representative images of wound healing assay and statistic results on cell wound closure in RKO-MCS-sc cells, RKO-MCS-shARHAP29 cells, RKO-MCS-sc cells treated with LiCl (0.5 μM) and RKO-MCS-shARHAP29 cells treated with LiCl (0.5 μM) were shown. N=3, **p<0.01, ***p<0.001. Scale bar: 50 μm. **D & E.** Representative images of trans-well assay and statistic results on migrated cells in RKO-MCS-sc cells, RKO-MCS-shARHAP29 cells, RKO-MCS-sc cells treated with LiCl (0.5 μM) and RKO-MCS-shARHAP29 cells treated with LiCl (0.5 μM) were

shown. N=3, **$p<0.01$, ***$p<0.001$. Scale bar: 50 μm. **F.** Western bolt to detect the expression of p-GSK3β, GSK3β, ZO-1, E-cadherin, Claudin-1, Vimentin, N-cadherin, β-catenin, ZEB1, Slug and Snail in RKO-MCS-sc cells, RKO-MCS-shARHAP29 cells, RKO-MCS-sc cells treated with LiCl (0.5 μM) and RKO-MCS-shARHAP29 cells treated with LiCl (0.5 μM), β-actin as loading control.

Figure S4

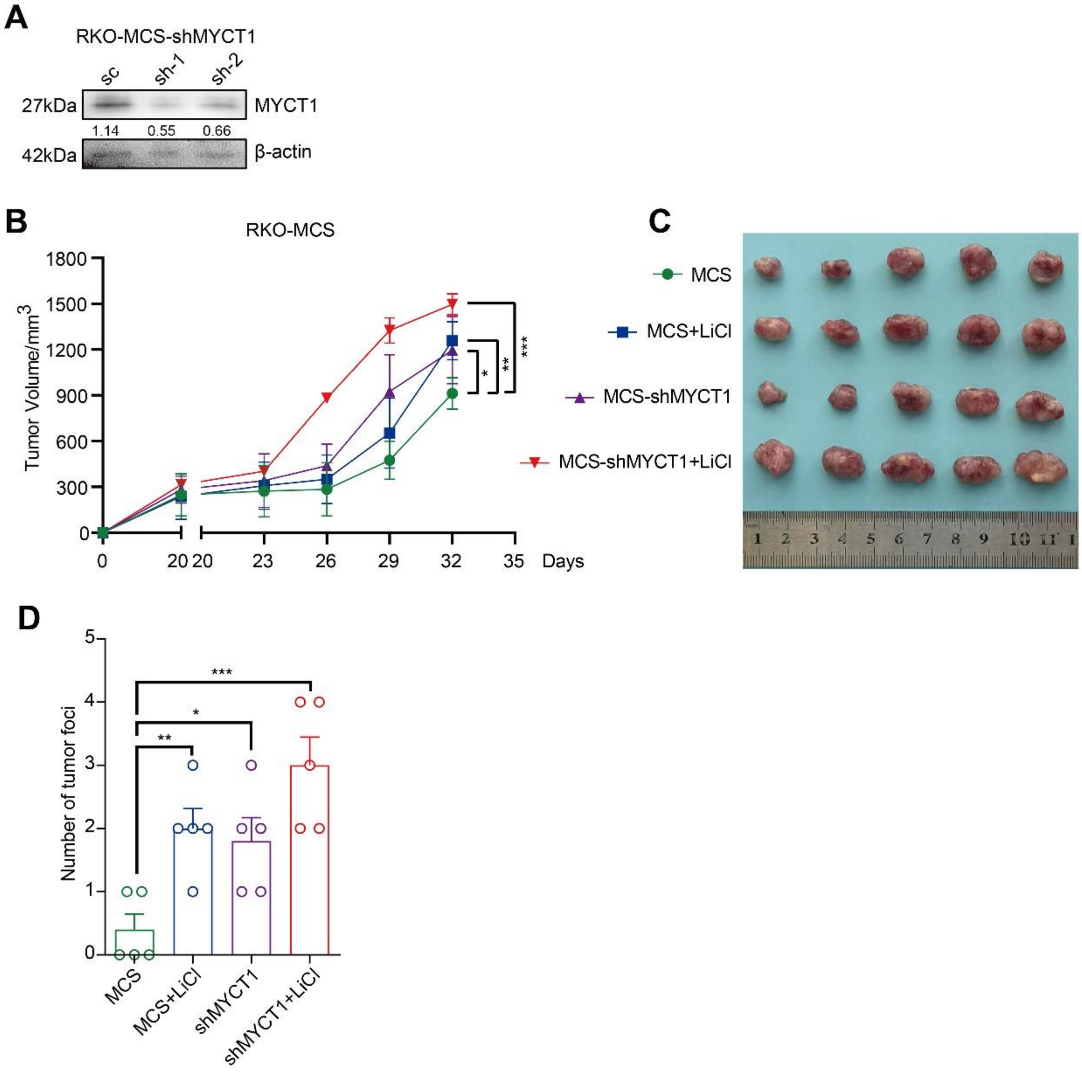

Figure 8. Combined MYCT1-shRNA and LiCl treatment inhibit CRC tumor metastasis and progression inhibition *in vivo.* A. Western blot to analysis MYCT1 knockdown efficiency in RKO-MCS cells. **B.** Nude mice were injected with stable RKO-MCS or RKO-MCS-shMYCT1 cells at the forth fat pad, then treated or not treated with LiCl, the tumor growth curves were measured. N=5, *p<0.05, **p<0.01, ***p<0.001. **C.** The tumors separated from different group mice were shown. **D.** Statistic results of metastatic nodules in the lungs from various groups were shown. N=5, *p<0.05, **p<0.01, ***p<0.001.

Table S1. Patients' information

Sample Number	ID Number	Sex	Age	Organ	Pathology Diagnosis	Type	Sampling Time	Blood
#1	3001342188	M	77	Colon	Adenocarcinoma	Malignant	25th May, 2018	No
#2	3001421844	F	83	Colon	Adenocarcinoma	Malignant	12th Oct, 2018	No
#3	3001630886	F	68	Colon	Adenocarcinoma	Malignant	12th Dec, 2018	No
#4	3001302212	F	67	Colon	Adenocarcinoma	Malignant	19th Jun, 2018	No
#5	3001693217	M	53	Colon	Adenocarcinoma	Malignant	30th Nov, 2018	No
#6	3000998773	M	80	Colon	Adenocarcinoma	Malignant	3th Mar, 2018	No
#7	3000645031	M	41	Colon	Adenocarcinoma	Malignant	4th Mar, 2018	No
#8	1004343219	M	62	Colon	Adenocarcinoma	Malignant	23th Jan, 2018	No
#9	3000702917	F	55	Colon	Colon	Adenocarcinoma	5th Sep, 2017	No
#10	3000649324	M	60	Colon	Colon	Adenocarcinoma	8th Sep, 2017	No

Table S2. Antibodies List

Antibody		Clone, Cat #	Vendor	City, State, Country
TBX21	Rabbit monoclonal	5214	Cell Signal Technology	Danvers, MA, USA
GSK3β	Rabbit monoclonal	12456	Cell Signal Technology	Danvers, MA, USA
p- GSK3β	Rabbit monoclonal	5558	Cell Signal Technology	Danvers, MA, USA
ARHGAP29	Rabbit monoclonal	12583-1-AP	Proteintech	Wuhan, China
β-actin	Mouse monoclonal	sc-47778	Santa Cruz Biotechnology	Santa Cruz, CA, USA
Epithelial-Mesenchymal Transition (EMT) Antibody Sampler Kit	Rabbit monoclonal	9782	Cell Signal Technology	Danvers, MA, USA
MYCT1	Rabbit polyclonal	22004-1-AP	Proteintech	Wuhan, China

Table S3.   Primer sequences

Name	Sequence
TBX21-shRNA-1	AAAAGCGCCAGGAAGTTTCATTTGGTTGGATCCAACCAAATGAAACTTCCTGGCGC
TBX21-shRNA-2	AAAAGGGAGAACTTTGAGTCCATGTTTGGATCCAAACATGGACTCAAAGTTCTCCC
ARHGAP29-shRNA-1	AAAAGCATCAGGTCAACTCTCTACTTTGGATCCAAAGTAGAGAGTTGACCTGATGC
ARHGAP29-shRNA-2	AAAAGCTCTAACAGTGCAGATATAATTGGATCCAATTATATCTGCACTGTTAGAGC
MYCT1-shRNA-1	AAAAGCTGTACTACAAAGAGATAGATTGGATCCAATCTATCTCTTTGTAGTACAGC
MYCT1-shRNA-2	AAAAGGAACAAGCAAATTCCTTTCCTTGGATCCAAGGAAAGGAATTTGCTTGTTCC
Sc control	AAAAGCTACACTATCGAGCAATTTTGGATCCAAAATTGCTCGATAGTGTAGC
CHIP-F1	TCATGGAAGGAGAATATGACCTT
CHIP-R1	AGCAATCATCCTTTCATGTAACAG
CHIP-F2	TATCTAGAAAATACTAGAGCCAGG
CHIP-R2	CCTTCCATGACATCACCTTC
CHIP-F3	GTCCCAGCTACATGGGT
CHIP-R3	TTTCTAGATATGTGACCAGTACA

CHIP-F4	TCTCACTTATACTTTGGAAGGGTTT
CHIP-R4	TAGCTGGGACTCCAGGGG
CHIP-F5	GTTTTGCTGGGGGATG
CHIP-R5	TAAGTGAGAAGCTATCATGCTCT
